# Metastatic nonpalpable invasive lobular breast carcinoma presenting as rectal stenosis: a case report

**DOI:** 10.1186/s13256-015-0568-x

**Published:** 2015-04-24

**Authors:** Tadatoshi Osaku, Hideaki Ogata, Shunsuke Magoshi, Yorichika Kubota, Fumi Saito, Shinsaku Kanazawa, Hironori Kaneko

**Affiliations:** Department of Surgery, Division of Breast and Endocrine Surgery (Omori), Toho University School of Medicine, 6-11-1 Omori-Nishi, Ota-ku, Tokyo 143-8451 Japan

**Keywords:** Invasive lobular carcinoma, Carcinomatous peritonitis, Breast cancer

## Abstract

**Introduction:**

Invasive lobular carcinomas have an increased propensity for distant metastases, particularly to the peritoneum, ovaries, and uterus. In contrast, distant metastases of nonpalpable lobular carcinomas are extremely rare, and the causes of underlying symptoms of primary carcinomas remain unclear. We report a case of an asymptomatic invasive lobular carcinoma with a primary mammary lesion in a patient with rectal stenosis.

**Case presentation:**

A 69-year-old Japanese woman presented to our hospital for treatment of constipation. Although rectal stenosis was confirmed, thorough testing of her lower digestive tract did not identify its cause. Thus, an exploratory laparotomy and tissue biopsy was performed, and the presence of an invasive lobular carcinoma was confirmed. Subsequent breast examinations showed that the invasive lobular carcinoma that led to the rectal stenosis was a metastatic lesion from a primary lesion of the breast duct. As the present breast lobular carcinoma was asymptomatic and nonpalpable, we did not initially consider metastatic breast cancer as a cause of her symptoms, and the final diagnosis was delayed.

**Conclusions:**

Peritoneal metastasis from nonpalpable invasive lobular carcinomas is very rare. However, breast cancer metastasis should be considered when carcinomatous peritonitis is present in a patient with an unknown primary cancer.

## Introduction

Compared with invasive ductal carcinomas, invasive lobular carcinomas have an increased propensity for distant metastases, particularly in the peritoneum, ovaries, and uterus [1-4]. However, although such metastases are rare for small asymptomatic lesions, the presence of a distant metastatic invasive lobular carcinoma may reflect a detectable primary breast lesion, as shown for invasive carcinomas [4-6]. Therefore, such metastases can be traced back to their primary lesions with relative ease. However, if unnoticed, primary carcinomas are not easily identified as the cause of underlying symptoms.

In our case report, asymptomatic, nonpalpable invasive lobular carcinoma from a primary breast lesion was discovered, following assessments of the symptoms caused by rectal stenosis. Prior to suspicion of a primary breast lesion, investigations were primarily focused on our patient’s lower digestive tract, and a final diagnosis was made only after an exploratory laparotomy. Herein, we describe diagnostic strategies and suggest practices that may avoid diagnostic pitfalls during initial evaluations of symptoms.

## Case presentation

**Figure 1:**
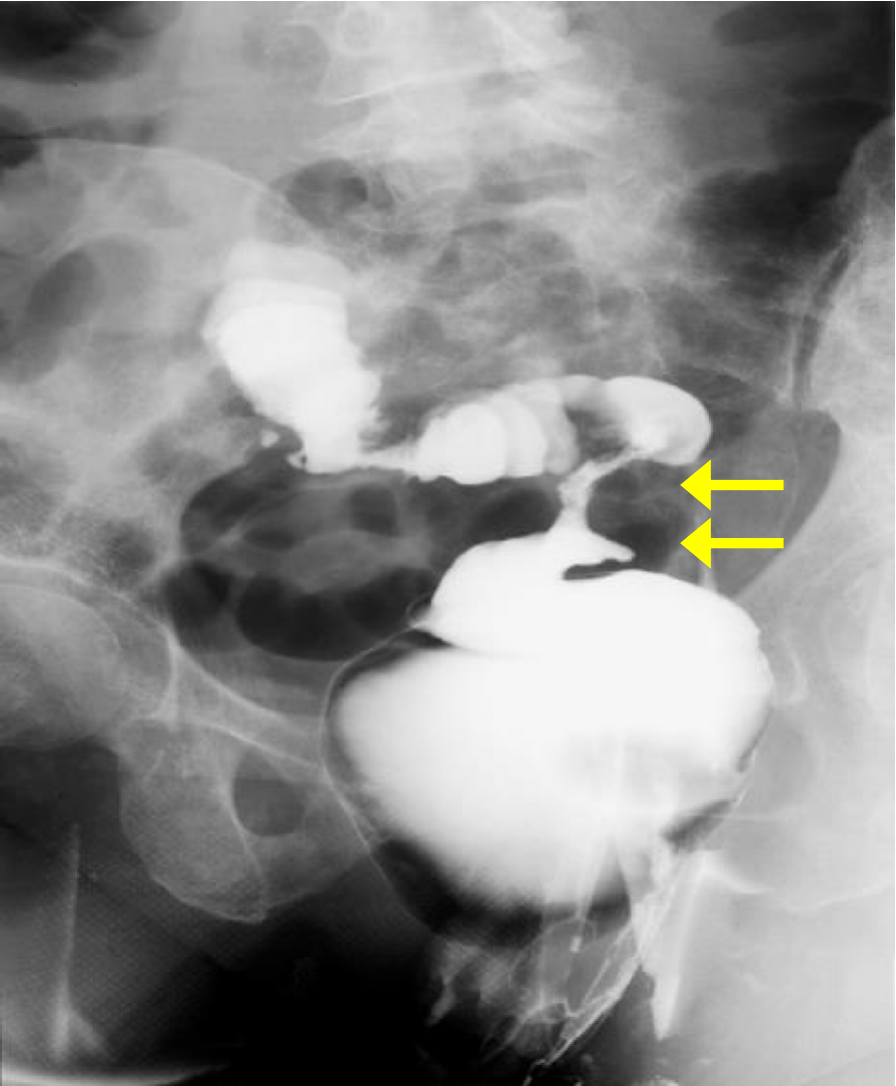
A barium enema image showing rectosigmoid stenosis but no mucosal abnormalities. Arrows show locations of stenosis.

A 69-year-old Japanese woman with unremarkable personal and family medical histories presented to the general surgery department of our hospital with constipation for approximately one month. Her rectal examination revealed rectal stenosis and possible rectal cancer. Her subsequent barium enema examination showed rectosigmoid stenosis (Figure 1), but no irregularities in her mucosa. Accordingly, a biopsy specimen was taken from her mucosa by lower abdominal gastrointestinal endoscopy, and showed no evidence of cancer cells in the area of the stenosis. Her blood tests revealed slightly elevated levels of the cancer antigens carbohydrate antigen 15-3 (CA15-3) and cancer antigen 125 (CA12-5), but all other values were within normal ranges.

**Figure 2:**
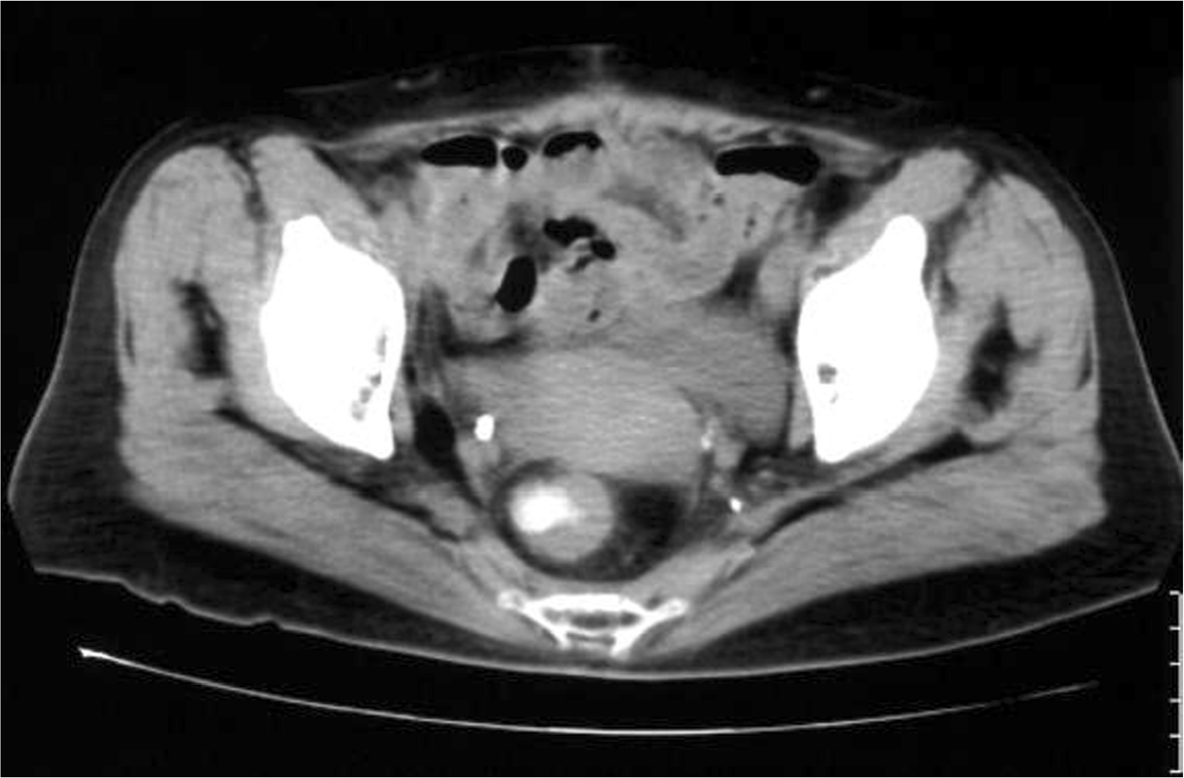
An abdominal computed tomography image showing rectal stenosis with thickening of the rectosigmoid wall.

Apart from thickening of her rectosigmoid wall in the stenotic region (Figure 2), no other abnormalities were detectable in her abdominal and chest computed tomography (CT) scans. Full-length positron emission tomography (PET) was suggested as a non-invasive procedure for detecting the primary lesion, but she declined for financial reasons. Hence, an exploratory laparotomy was performed to identify the cause of her rectosigmoid stenosis.

**Figure 3:**
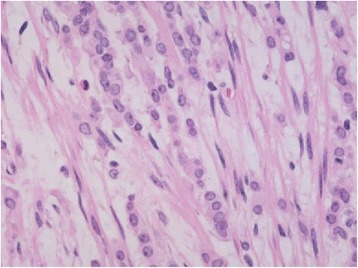
Histopathological findings of a peritoneal sample. The Indian file pattern of small cells (hematoxylin and eosin, ×400 magnification) suggests peritoneal metastasis from an invasive lobular carcinoma of the breast.

Her surgical examinations revealed nodules dispersed throughout her abdominal cavity, and additional stenosis in her rectum and ileocecum, suggesting peritoneal metastasis. Histopathological findings from a biopsy specimen of the peritoneal material from around the rectosigmoid region were consistent with a diagnosis of peritoneal metastasis of an invasive lobular carcinoma, likely from the breast (Figure 3). However, subsequent breast examinations by the breast oncology department revealed no visible or palpable abnormalities or secretions in either of her breasts.

**Figure 4:**
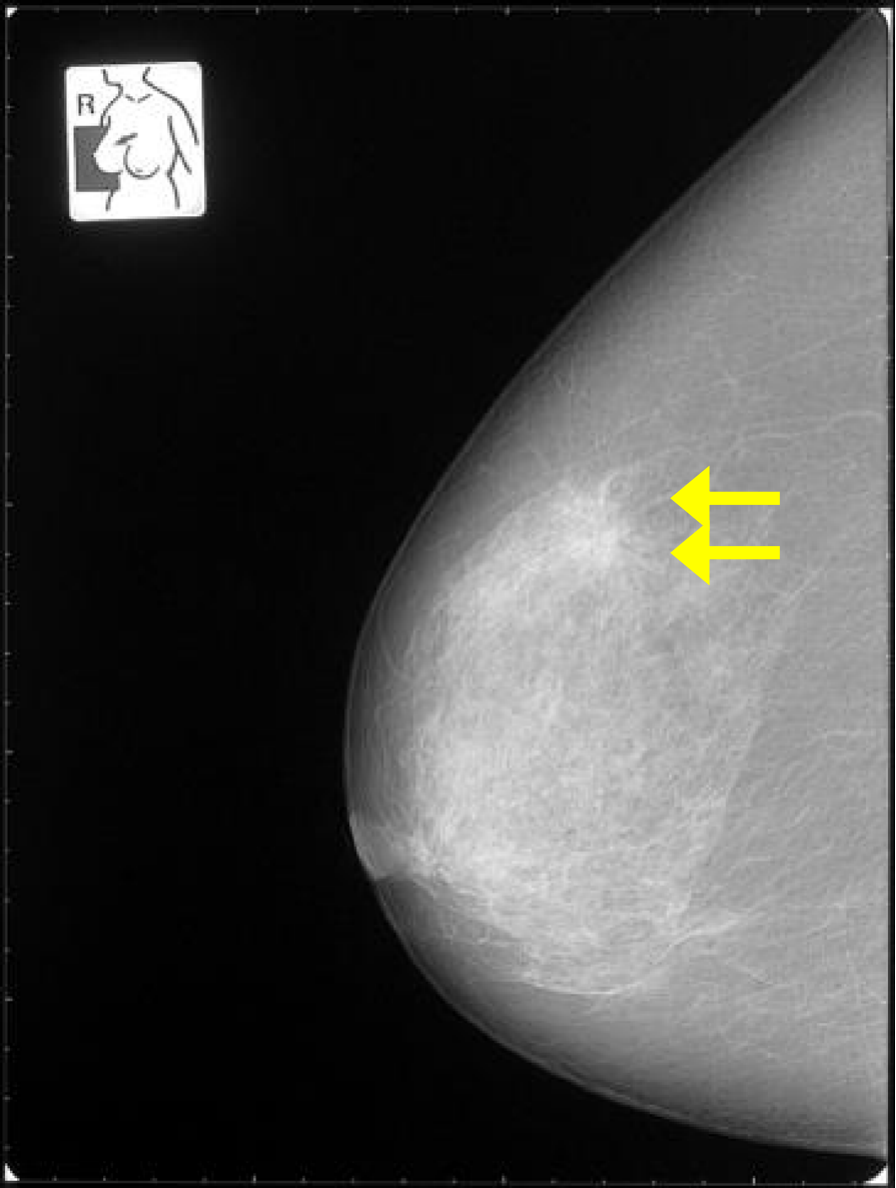
Mammogram showing abnormal tissue in the right breast (category four). Arrows indicate abnormal tissue.

**Figure 5:**
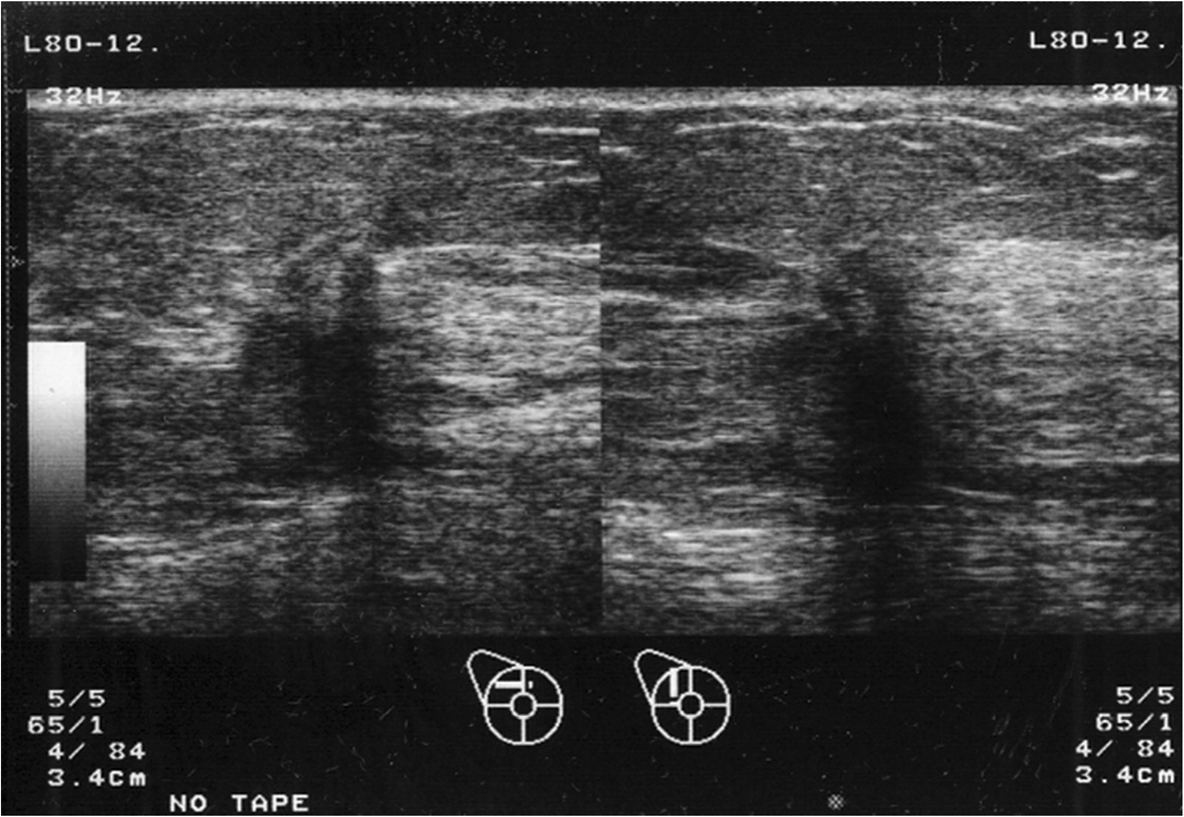
A ductal ultrasonogram showing an irregularly shaped mass of approximately 5 × 6mm with unclear boundaries in area C of our patient’s right breast. The internal echogenicity was uneven and the posterior echo was dissipated.

Subsequent mammogram images showed a spiculated structure of lactiferous ducts in area C of her right breast (Figure 4). Moreover, a ductal ultrasonography revealed a lesion of approximately 5 × 6mm in the same area (Figure 5). A subsequent biopsy specimen of the duct showed cells with histopathological characteristics similar to those in her peritoneal nodules.

Therefore, the final diagnosis was of rectal stenosis caused by carcinomatous peritonitis arising from a metastatic invasive lobular breast cancer.

After her diagnosis, courses of hormone therapy and taxane- and anthracycline-based drugs were administered. Her rectal stenosis symptoms improved immediately after completion of two courses of weekly paclitaxel, and she was able to start ingestion. After discharge, a sequential regimen of paclitaxel, docetaxel, and anthracycline was administered in the out-patient department of our institution for one year, followed by hormone therapy for three years. However, the disease gradually progressed and she died four years after the initial diagnosis.

## Discussion

Distant metastases occur more frequently from lobular carcinomas than from more common invasive breast cancers. Furthermore, invasive lobular carcinomas metastasize to the peritoneum and retroperitoneum in 93% of cases (including autopsies), the ovaries in 36% of cases, and the uterus in 43% of cases [1], and can metastasize to other areas and organs of the digestive tract [2-4]. However, such distant metastases are rare in the absence of palpable primary lesions.

Although it is well known that invasive lobular carcinoma tends to metastasize to the peritoneum, patients with initial symptoms that are exclusively abdominal are rare. We performed PubMed searches using the search terms ‘invasive lobular carcinoma’, ‘peritoneum’, ‘metastatic’, ‘unknown origin’, and ‘occult breast cancer’. The resulting studies showed only a few cases in which initial symptoms in the retroperitoneum and digestive tract were caused by metastasis of palpable invasive lobular carcinomas [5,6], and some recurrent cases had reported involvement of symptoms in digestive organs more than 10 years postoperatively [7,8]. However, only two case studies reported the appearance of initial symptoms in the digestive tract due to metastasis of nonpalpable invasive lobular carcinomas [9,10]. One of these cancers metastasized to the stomach and the other metastasized to the colon, and both cases were diagnosed preoperatively, following identification of adenocarcinoma in endoscopic specimens from mucosal lesions with invasions of lobular carcinoma.

In our case report, a biopsy specimen of our patient’s associated mucosa showed no evidence of cancer cells in the area of her stenosis. This negative result reflected the presence of a specific metastatic form of invasive lobular carcinoma known as a peritoneal metastasis, which reportedly invades perirectal areas and causes rectal stenosis, but does not reach the mucosal layer. Moreover, pathological analyses of perirectal tissue specimens from her laparotomy showed invasive lobular carcinoma, as indicated by lobular carcinoma histology showing a non-polarized, even distribution of small cancer cells that had diffusely invaded her interstitial tissue. These cells typically appear in a single row, which is often referred to as an Indian file [11,12], and usually form a solid nest or occasionally a signet-ring shape, but rarely a glandular structure. Intercellular mucus is also frequently present. Estrogen receptor (ER) and progesterone receptor (PgR) expression are more frequent in lobular carcinomas than in other invasive carcinomas [13], although human epidermal growth factor receptor 2 (HER2) overexpression is rare [11]. Furthermore, E-cadherin gene mutations are present in more than 85% of invasive lobular carcinomas, likely reflecting characteristically low protein expression of E-cadherin [14,15]. Our patient’s pathological examination revealed characteristics of an invasive lobular carcinoma, including a row of small cells (Indian file), negative staining for E-cadherin and HER2, and positive staining for both ER and PgR. However, the clinicopathological mechanisms that induced this metastasis from a non-palpable cancer are unclear.

In our case report, extensive initial assessments failed to reveal the cause of our patient’s intestinal stenosis, and a conclusive diagnosis was only made after a biopsy during an exploratory laparotomy. PET/CT imaging has proven utility as an imaging modality for visceral metastases, and it might have been effective in this case. However, despite its high accuracy in breast cancer staging, PET/CT was not deemed cost-effective for this initial evaluation of breast cancer [16], according to the absence of financial support for the procedure from the national health care system in Japan.

In retrospect, a tentative diagnosis could have been made according to the guidelines for unknown primary neoplasms. Specifically, the 2012 National Comprehensive Cancer Network (NCCN) Guidelines for Diagnosis of Unknown Primary Cancers recommend a comprehensive review of medical histories for patients in whom such cancers are suspected, including careful examination of mammary, urinary, and reproductive tissues. Following identification of the potential location of the primary lesion by biopsy, further testing using mammography should be performed to confirm tentative findings [17].

As the present breast tumor was non-palpable, visual or palpable confirmation of abnormalities in our patient’s breast tissue was not immediately possible. However, a mammography or ultrasonography upon admission may have limited the physical trauma of the laparotomy, and would have warranted treatment earlier. Thus, primary breast lesions should be considered as possible underlying sources of invasive lobular carcinomas in cases of unknown primary cancer. Subsequently, noninvasive tests for breast cancers are warranted as a part of routine clinical assessments to determine the presence of such lesions.

## Conclusions

We report a case of nonpalpable invasive lobular carcinoma presenting with symptoms that were suggestive of carcinomatous peritonitis. Although peritoneal metastasis from a nonpalpable breast lesion is rare, breast cancer metastasis should be considered as a potential cause of carcinomatous peritonitis in patients with unknown primary cancers.

## Consent

Written informed consent was obtained from the patient’s next of kin for publication of this case report and all accompanying images. A copy of the written consent is available for review by the Editor-in-Chief of this journal.

## Abbreviations

CA12-5: Cancer antigen 125
CA15-3: Carbohydrate antigen 15-3
CT: Computed tomography
PET: Positron emission tomography
ER: Estrogen receptor
PgR: Progesterone receptor
HER2: Human epidermal growth factor receptor 2
NCCN: National Comprehensive Cancer Network
